# Surface Engineering of Bioactive Coatings for Improved Stent Hemocompatibility: A Comprehensive Review

**DOI:** 10.3390/ma16216940

**Published:** 2023-10-29

**Authors:** Amisha S. Raikar, Sushma Priya, Shilpa P. Bhilegaonkar, Sandesh N. Somnache, Deepak M. Kalaskar

**Affiliations:** 1 Department of Pharmaceutics, PES Rajaram and Tarabai Bandekar College of Pharmacy, Ponda 403401, India; shilpabhilegaonkar@gmail.com; 2 University College of London, Division of Surgery and Interventional Science, Royal National Orthopaedic Hospital, Rowland Hill Street, London NW3 2PF, UK; sushma.priya.21@ucl.ac.uk; 3 Department of Biomedical Engineering, Regenerative Medicine and Stem Cell (RMS) Labs, Indian Institute of Technology, Hyderabad 502285, India; 4 Department of Pharmaceutics, SSPM’s VP College of Pharmacy, Madkhol 416510, India; sandeshsomnache@gmail.com

**Keywords:** surface engineering, bioactive coatings, stent, hemocompatibility, thrombogenic

## Abstract

Cardiovascular diseases continue to be a major contributor to illness and death on a global scale, and the implementation of stents has given rise to a revolutionary transformation in the field of interventional cardiology. The thrombotic and restenosis complications associated with stent implantation pose ongoing challenges. In recent years, bioactive coatings have emerged as a promising strategy to enhance stent hemocompatibility and reduce thrombogenicity. This review article provides an overview of the surface engineering techniques employed to improve the hemocompatibility of stents and reduce thrombus formation. It explores the mechanisms underlying thrombosis and discusses the factors influencing platelet activation and fibrin formation on stent surfaces. Various bioactive coatings, including anticoagulant agents, antiplatelet agents, and surface modifications, are discussed in detail, highlighting their potential in reducing thrombogenicity. This article also highlights a multitude of surface modification techniques which can be harnessed to enhance stent hemocompatibility including plasma treatment, physical vapor deposition (PVD), chemical vapor deposition (CVD), and electrodeposition. These techniques offer precise control over surface properties such as roughness, charge, and composition. The ultimate goal is to reduce platelet adhesion, tailor wettability, or facilitate the controlled release of bioactive agents. Evaluation methods for assessing hemocompatibility and thrombogenicity are also reviewed, ranging from in vitro assays to animal models. Recent advances in the field, such as nanotechnology-based coatings and bioactive coatings with controlled drug release systems, are highlighted. Surface engineering of bioactive coatings holds great promise for enhancing the long-term outcomes of stent implantation by enhancing hemocompatibility and reducing thrombogenicity. Future research directions and potential clinical applications are discussed, underscoring the need for continued advancements in this field.

## 1. Introduction

**Figure 1:**
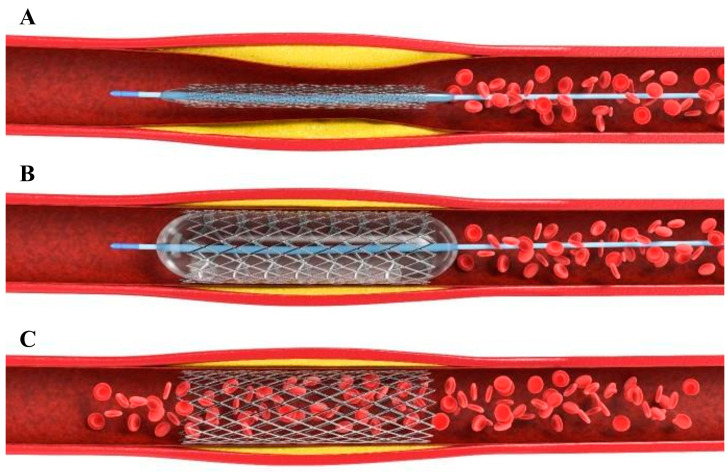
Schematic representation of a stent used in angioplasty, demonstrating its role in improving blood flow through the affected artery. (**A**) The stent is positioned at the site of the plaque. (**B**) The balloon is filled with fluid and, as a result, the stent expands, displacing the plaque. (**C**) The balloon is depressurized and extracted through the flexible tube.

Cardiovascular disease (CVD) refers to a group of conditions that affect the heart and blood vessels, which can lead to serious health problems. The most common types of CVD include coronary artery disease (CAD), heart failure, stroke, and hypertension (high blood pressure). Atherosclerosis, hypertension, smoking, poor diet, and inactivity are regarded as primary causes of CVD. Symptoms include chest pain, shortness of breath, fatigue, and swelling. Lifestyle changes, medication, procedures like angioplasty or bypass surgery, and implantable devices are some of the interventions currently available to treat CVD. Cardiovascular diseases, including coronary artery disease, remain a primary cause of global mortality. Stents, small, tube-like medical devices typically made of metal or fabric that are used to support and open narrowed or blocked blood vessels or other tubular structures within the body, have revolutionized the treatment of these conditions by restoring blood flow through the affected arteries. These tiny mesh-like devices are implanted during an angioplasty procedure to mechanically open narrowed or blocked arteries and provide structural support to keep them open, as shown in Figure 1. Stents have prominently improved patient outcomes and reduced the need for more invasive surgical interventions. Percutaneous transluminal coronary angioplasty (PTCA) is a popular procedure for treating occlusive blood vessel disorders [1]. Each year, the number of treatments performed has increased, and it has been discovered that the procedure decreases the occurrence of restenosis, or the re-obstruction of the targeted artery. Restenosis is reduced due to the stent’s scaffolding effect, which inhibits elastic rebound and constrictive remodeling of the artery.

Stent implantation comes with certain challenges. Two major issues that can arise post-implantation are thrombosis and restenosis. Thrombosis pertains to the development of blood clots on the surface of the stent, which can cause sudden blockage of the blood vessel, potentially resulting in life-threatening situations like heart attack or stroke [2]. Restenosis, on the other hand, is the recurrence of artery narrowing due to excessive tissue growth at the stent site, reducing blood flow and necessitating further interventions [3].

To address these challenges, extensive research has focused on developing bioactive coatings for stents which are designed to enhance stent hemocompatibility and reduce thrombogenicity [4]. These coatings, when applied to the stent’s surface, interact favorably with blood components, promoting improved biocompatibility and reducing the risk of adverse reactions. Two novel advancements in this technology demonstrate promise in addressing thrombosis and in-stent restenosis [2,3]. One approach to improving stent biocompatibility is modifying their surfaces with materials that have a lower likelihood of inducing blood clotting and inflammation. These outer layers comprise inorganic elements such as carbon or silicon carbide, along with biomimetic substances like surfaces modified with phosphorylcholine [4]. An alternative effective approach, which has demonstrated a reduction in the proliferation of smooth muscle cells (SMCs) and a significant delay in in-stent restenosis, involves applying a coating on stents containing therapeutic agents like Rapamycin or Taxol. These drugs are then gradually released at the site of implantation [5]. Usually, these therapeutic agents are integrated into a polymer-based framework. Early investigations utilizing stents coated with biodegradable polymers, such as polyglycolic acid/polylactic acid copolymers, polycaprolactone polyhydroxy (butyrate valerate), and poly (ethylene oxide)/polybutylene terephthalate (PEO/PBTP), as well as nonbiodegradable polymers like polyurethane (PUR), silicone (SIL), and poly (ethylene terephthalate) (PETP), yielded unsatisfactory outcomes, suggesting that the presence of these polymers triggered prolonged inflammatory responses [6,7]. As a result, ongoing research has focused on developing improved coatings with reduced inflammatory reactions and more precise drug release mechanisms to enhance the efficacy and long-term performance of stents in clinical applications.

This review focuses on the primary objective of offering a comprehensive overview of bioactive coatings for stents and their potential to enhance hemocompatibility and mitigate thrombogenicity. It also summarizes the current state of research and development in the field, highlighting key advancements, challenges, and future directions. By critically evaluating the existing literature and studies, the objective is to provide valuable insights for researchers, clinicians, and industry professionals working on improving stent technologies. The ultimate goal is to contribute to advancing the creation of stents that are both safer and more effective, reducing complications and improving patient outcomes in the treatment of cardiovascular diseases.

### Physiological Process of Hemostasis and the Formation of Blood Clots

**Figure 2:**
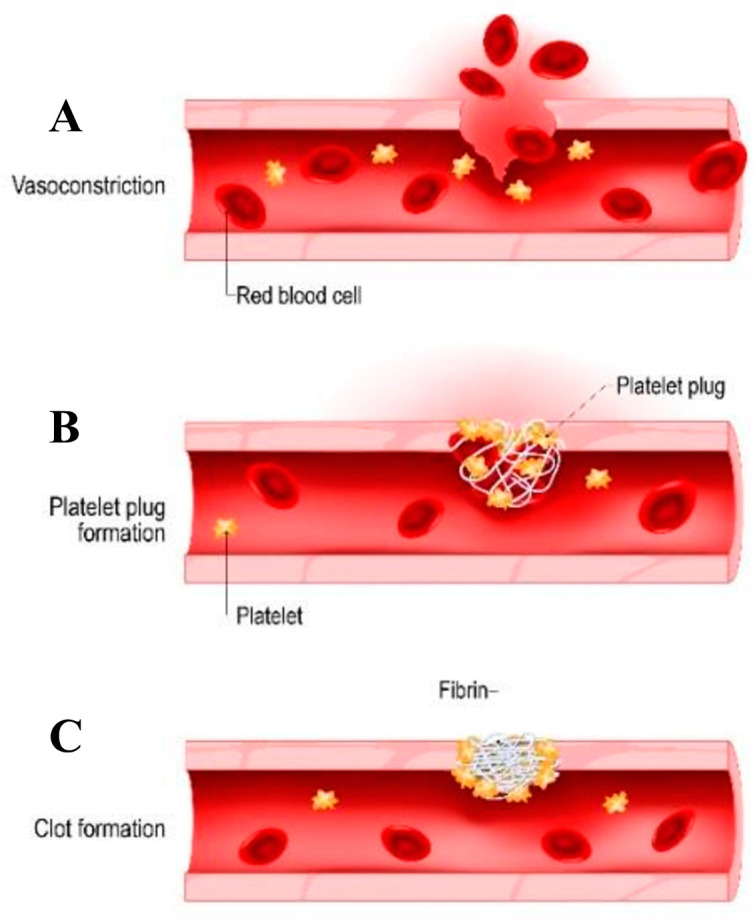
Illustration depicting the process of hemostasis, showcasing the sequence of events that occurs to control bleeding upon vessel injury. (**A**) Vasoconstriction to reduce bleeding (**B**) Platelet sticks together to form platelet plug (**C**) Blood proteins and platelets form a stable clot to stop bleeding.

Hemostasis refers to the natural physiological process through which the body initiates the formation of a blood clot to control and prevent excessive bleeding when a blood vessel is damaged. This intricate process involves a series of interactions and reactions that are triggered in response to injury [8]. The hemostasis process can be categorized into three main phases: vascular constriction, platelet plug formation, and blood coagulation/clot formation, as depicted in Figure 2. Continuous research aims to develop a deeper understanding of these processes and explore novel therapeutic interventions for improved hemostatic control.

Hemostasis is a crucial process activated in response to vascular injury to prevent excessive blood loss and maintain the integrity of blood vessels. The initial step in hemostasis is vascular constriction, wherein the smooth muscle within the vessel wall undergoes contraction, leading to the narrowing of the injured blood vessel (Figure 2A). This vasoconstriction helps reduce blood flow to the injured area, aiding in limiting blood loss [8]. Vascular constriction is mediated by neural reflexes, local chemical factors, and the release of endothelin, a vasoconstrictor peptide platelet, which are tiny non-nucleated cellular fragments originating from megakaryocytes that have a crucial function in hemostasis by creating the initial plug that aids in blood clotting. Following a vascular injury, platelets attach themselves to the exposed collagen and von Willebrand factor (vWF) present in the subendothelial tissue. This attachment is made possible by glycoprotein Ib (GpIb), the primary receptor for vWF. The adhered platelets undergo alterations in shape, increasing their surface area and forming numerous pseudopods. Subsequently, platelets undergo degranulation, releasing various substances from their alpha (α) and dense (δ) granules. Among other components, these substances encompass factors like P-selectin, fibrinogen, fibronectin, factor V, factor VIII, platelet factor IV, platelet-derived growth factor, and serotonin. During degranulation, calcium is also released, binding to phospholipids exposed due to platelet activation [9]. Platelet aggregation is further stimulated by the production of thromboxane A2 (TxA2) and adenosine diphosphate (ADP) during platelet activation [8,10]. During the initial phase of hemostasis, platelets, the vessel wall, and adhesive proteins interact, leading to the creation of an initial “platelet plug”. The inner lining of blood vessels, composed of endothelial cells, displays antithrombotic properties due to various components, including negatively charged heparin-like glycosaminoglycans, neutral phospholipids, the synthesis and release of platelet inhibitors, coagulation inhibitors, and fibrinolysis activators [8]. Below the endothelium lies the subendothelial layer, which is highly thrombogenic and contains substances like collagen, Von Willebrand factor (vWF), laminin, thrombospondin, and vitronectin, playing a crucial role in platelet adhesion [11]. Collagen is a key component in the process of blood clotting. When blood vessels are damaged, exposed collagen triggers platelet adhesion and activation. Platelets stick to collagen, change shape, and form clumps to temporarily stop bleeding. Thromboxane A2 and ADP work together to enlarge the platelet aggregate, resulting in the formation of the platelet plug, which acts as a temporary seal for the vascular injury (Figure 2B). The binding of ADP also triggers a structural change in the glycoprotein IIb/IIIa (GpIIb/IIIa) receptors on the platelet surface, leading to the deposition of fibrinogen. Thrombin, produced during this process, catalyzes the conversion of fibrinogen to fibrin, enhancing the stability of the platelet plug, now referred to as secondary hemostasis [8]. In contrast, prostacyclin inhibits platelet aggregation, maintaining a balance with thromboxane A2. This localized platelet aggregation prevents the clot from extending and ensures the patency of the vessel lumen. The final stage of hemostasis is blood coagulation or clotting, which involves a complex cascade of reactions that convert soluble proteins in the blood, known as clotting factors, into an insoluble protein called fibrin, the details of which are given in Table 1. Fibrin forms a mesh-like structure that strengthens the platelet plug and traps red blood cells, ultimately leading to the formation of a stable blood clot (Figure 2C).

The clotting cascade is initiated at the injury by the presence of tissue factor (TF). TF interacts with clotting factor VII, setting off a sequence of enzymatic reactions involving several clotting factors [14]. These reactions occur in a sequential and controlled manner, forming a cascade that involves transforming fibrinogen, a soluble protein, into fibrin. Fibrin strands interweave with the platelet plug and form a stable clot, reinforcing the seal over the injured vessel. The clotting process is regulated by a delicate balance of procoagulant and anticoagulant factors to prevent excessive clot formation (thrombosis) or uncontrolled bleeding. After the damaged blood vessel has healed, the clot undergoes a gradual dissolution process known as fibrinolysis. During fibrinolysis, an enzyme called plasmin breaks down the fibrin strands, enabling the restoration of normal blood flow.

**Figure 3:**
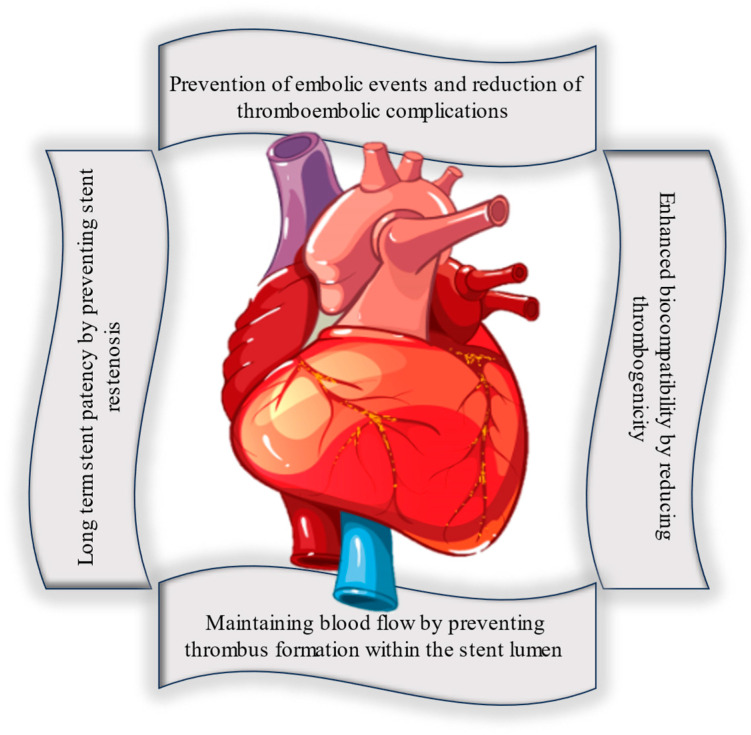
Key reasons for designing stent coatings to minimize platelet activation and fibrin formation.

Thrombosis on the surface of stents can occur due to multiple factors and mechanisms [15]. The presence of a foreign material (the stent) within the blood vessel can disrupt the normal hemostatic balance and trigger an excessive clotting response. Table 2 depicts key factors and mechanisms contributing to stent thrombosis:

To address these thrombogenic factors, various strategies have been employed; one approach involves using bioactive coatings on stents, which can release drugs or agents over time. These coatings are designed to inhibit platelet activation and clot formation around the stent. For instance, drug-eluting stents release medications that hinder the growth of smooth muscle cells and reduce inflammation, thereby decreasing the risk of restenosis, or re-narrowing of the treated artery. The patients who receive stents are often prescribed antithrombotic medications, such as antiplatelet drugs like aspirin and clopidogrel, to further reduce the risk of clot formation on the stent’s surface. Stent surfaces themselves can be modified to enhance biocompatibility and reduce clot formation risk, including smoother surfaces or specialized coatings that discourage platelet adhesion. Improvements in stent design, featuring thinner struts and better flexibility, aim to minimize blood flow disruption and reduce the potential for injury to the blood vessel wall, ultimately lowering the likelihood of clot formation. These approaches aim at minimizing platelet activation, promote rapid endothelialization, and maintain a healthy balance in the coagulation cascade to prevent stent thrombogenicity. Designing stent coatings to minimize platelet activation and fibrin formation is of paramount importance in preventing stent thrombosis and ensuring the long-term success of stent implantation. Figure 3 shows key reasons why this aspect is crucial.

## 2. Bioactive Coating

The use of bioactive coatings has surfaced as a favorable strategy to boost the hemocompatibility of stents, offering potential solutions to address the challenges associated with stent implantation. These coatings are specifically designed to interact favorably with blood components, leading to reduced thrombogenicity and improved biocompatibility.

Antiplatelet coatings are engineered to inhibit platelet activation and aggregation on the stent’s surface. By incorporating antiplatelet drugs, such as aspirin or clopidogrel, into the coating, a gradual release of these drugs inhibits platelet function. It works to prevent excessive platelet adhesion, thus reducing the risk of thrombus formation and ultimately improving the sustained stent patency Antithrombotic coatings aim to minimize clot formation by interfering with the clotting cascade. These coatings may contain anticoagulant drugs, like heparin or warfarin, or antithrombin agents that inhibit the activity of clotting factors or prevent fibrin formation. This approach significantly reduces the risk of thrombosis on the stent’s surface. Endothelialization-promoting coatings focus on accelerating the regeneration of the endothelial cell layer on the stent’s surface. Incorporating substances that mimic the extracellular matrix or promote endothelial cell attachment, growth, and migration, these coatings facilitate rapid endothelialization. A healthy endothelial lining contributes to reducing platelet adhesion, preventing thrombus formation, and minimizing the occurrence of restenosis. Anti-inflammatory coatings address the role of inflammation in the thrombotic response to stent implantation [23]. These coatings incorporate anti-inflammatory drugs or agents that help suppress the release of inflammatory mediators, reduce leukocyte activation and adhesion, and minimize platelet and clotting factor recruitment. By mitigating the inflammatory response, anti-inflammatory coatings contribute to improved hemocompatibility. Surface modification coatings alter the physical or chemical properties of the stent’s surface to improve its hemocompatibility. For example, hydrophilic coatings promote better blood compatibility by reducing platelet adhesion and activation [23]. Surface coatings with improved biocompatible materials or nanoscale modifications enhance the interaction between the stent and blood components, thus reducing the risk of thrombosis. The choice and combination of bioactive coatings depend on factors like stent design, intended application, and desired therapeutic outcomes.

In a study by Zhang et al., 2014 on patients with biodegradable polymer-coated drug-eluting stents, the researchers aimed to determine the optimal duration of dual antiplatelet therapy (DAPT) and its impact on bleeding events. They found that patients receiving DAPT for more than 6 months had a higher risk of bleeding compared to those on a 6-month regimen. Factors contributing to bleeding included age, diabetes, a history of coronary artery disease, and longer DAPT duration. Patients with bleeding also faced a greater risk of adverse cardiac events. Therefore, prolonged DAPT beyond 6 months after stent implantation may lead to increased bleeding and worse cardiac outcomes [24].

The study by Lin explored the potential antiplatelet activity of paclitaxel, a drug commonly used in coronary revascularization and in preventing in-stent restenosis. The findings indicated that paclitaxel effectively inhibited platelet aggregation triggered by collagen, a major contributor to in-stent restenosis, while having minimal impact on other platelet activators. It was observed that paclitaxel interfered with signaling molecules downstream of the collagen receptor glycoprotein VI (GPVI), ultimately reducing granule release and GPIIbIIIa activation. Paclitaxel also demonstrated antithrombotic effects without significantly affecting normal hemostasis. These results suggest that paclitaxel may offer additional benefits beyond its anti-proliferative properties when used on drug-coated balloons and drug-eluting stents for coronary revascularization and in preventing in-stent restenosis [25].

**Figure 4:**
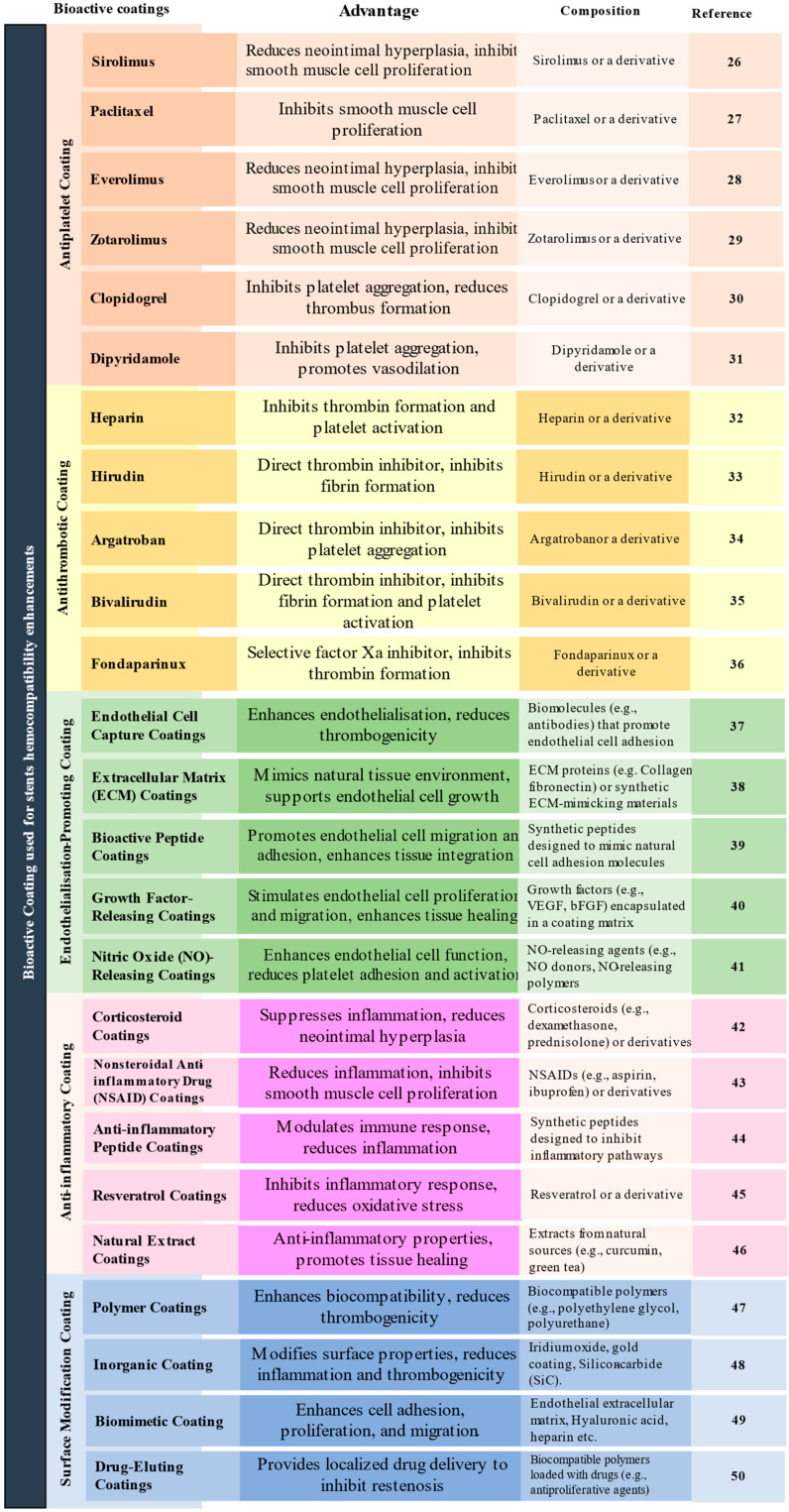
Different types of bioactive coatings used for stent hemocompatibility enhancements [26,27,28,29,30,31,32,33,34,35,36,37,38,39,40,41,42,43,44,45,46,47,48,49,50].

Many more such studies were conducted using different types of bioactive coatings for stent hemocompatibility enhancements, and these studies are depicted in Figure 4.

## 3. Drug-Eluting Stent (DES)

**Figure 5:**
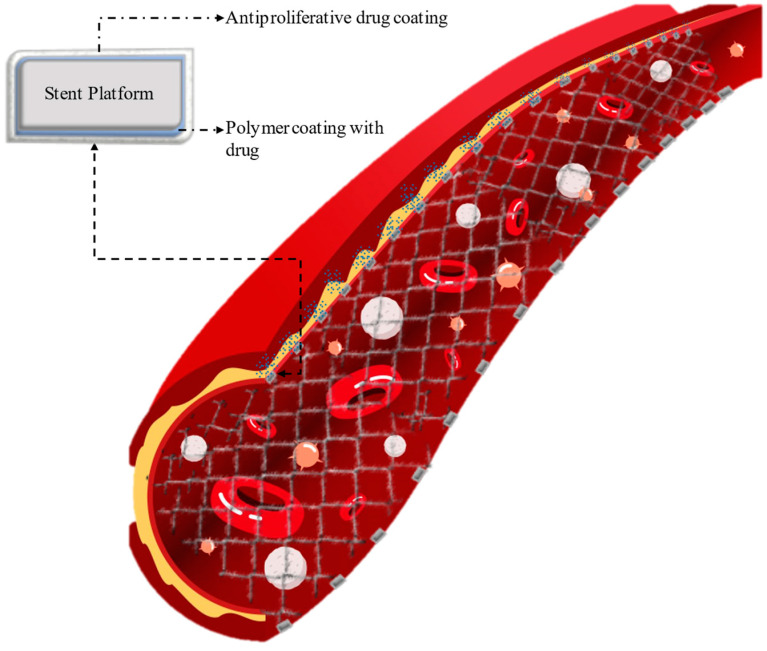
Systematic representation of a drug-eluting stent (DES).

Drug-eluting stents (DESs) are widely utilized in interventional cardiology practice to treat narrowed coronary arteries caused by arteriosclerosis, as depicted in Figure 5. The history of interventional cardiology dates back to 1977, when balloon angioplasty was introduced [49]. In 1986, the initial uncoated metallic stent (BMS) was launched by Sigwart et al. [24]. The first drug-eluting stent (DES) was introduced in Europe in 2002, and since then, numerous companies have offered a wide range of DES options to enhance the remediation of coronary artery disease. The initial study of drug-eluting stents (DESs) employed stainless-steel scaffolding covered with either sirolimus or paclitaxel. In the second generation, cobalt–chromium scaffolding with various polymer coatings was introduced, leading to improved flexibility, biocompatibility, and re-endothelialization [50]. The latest advancement in drug-eluting stents (DESs), known as the third generation, is currently undergoing clinical trials and involves the use of biodegradable polymers or completely bioabsorbable scaffolds.

DESs have proven to be superior to balloon angioplasty alone, which often results in restenosis. Early restenosis is caused by neointimal hyperplasia resulting from the movement and multiplication of vascular smooth muscle cells after stent deployment. Drug-eluting stents (DESs) were invented to address complications such as restenosis by incorporating anti-proliferative drugs that reduce cell proliferation within the stent. Although drug-eluting stents (DESs) have substantially decreased early restenosis rates, extended studies have unveiled a novel issue: stent thrombosis delayed greater than 30 days and extremely delayed greater than 12 months [51,52]. This complication is linked to the inhibitory impact of DES on cell proliferation, which retards the regrowth of endothelial cells over the stent’s surface. Upon discontinuation of oral antiplatelet therapy, uncovered scaffold material can trigger platelet activation, leading to late restenosis or thrombosis. Stent thrombosis is a life-threatening complication with infrequent occurrence with elevated fatality rate, making anticoagulation crucial after stent implantation. To address hypersensitivity to stents, polymer-free or biodegradable polymers have been introduced. The use of biodegradable stents has gained significance in promoting healthy long-term vasomotion. Each drug-eluting stent has unique characteristics with advantages and disadvantages based on factors such as the stent’s drug-carrying capacity, drug release pharmacokinetics, polymer integrity, biocompatibility, effect on the thinning of the vascular wall, potential for aneurysm formation, and susceptibility to delayed restenosis. The use of materials not naturally present in the human body has raised concerns about biocompatibility, leading to investigations into more biocompatible and degradable compounds.

Complications following stent implantation include stent occlusion, inflammatory response, neointimal hyperplasia, and scaffold fracture due to material fatigue. Stent fracture is often under-recognized, as it may not trigger noticeable symptoms, but it is a concern with risk factors including longer stent length and stents placed in certain locations. Manufacturers are required to demonstrate 10-year durability through stress testing, as mandated by the FDA [53]. Although most stent fractures without restenosis are conservatively managed and show good outcomes, malposition is another issue that can cause late stent thrombosis. It occurs in a small percentage of cases and refers to inadequate stent strut alignment with the vessel wall. Ensuring proper biocompatibility and addressing potential complications are ongoing challenges in improving the tolerability and potency of DESs for the treatment of CAD. The choice of drugs and their release kinetics can be tailored to optimize hemocompatibility and reduce thrombogenicity.

## 4. Inorganic Coatings

Inorganic coatings offer a range of potentially viable materials for the modification of medical implant surfaces, including stents. Some typically used inorganic treatment materials on stents include gold, silicon carbide, iridium oxide, and diamond-like carbon. These materials provide unique properties and characteristics that can enhance the performance and biocompatibility of stents.

### 4.1. Gold Coating

Previously, gold coating was a popular choice to improve the visibility of stainless-steel (SS) stents during fluoroscopy. It was especially useful for reducing stent thickness to a range of 50–80 μm. By having a radiopacity six times higher than steel, a mere 5 μm gold coating on both the sides effectively doubled and the X-ray visibility of an 80 μm thick steel stent.

In their investigation of the vascular behavior in pig coronary arteries, Edelman et al. conducted a study comparing standard gold plating with thermally treated gold coating [54]. The findings indicated that the thermally processed coating resulted in reduced neointimal hyperplasia and inflammation. This favorable outcome was attributed to the smoother gold surface and the elimination of impurities incorporated within the coating. The study underscored the significant influence of surface properties and material purity on interactions between tissues and materials.

In contrast to the promising preclinical results, clinical trials involving gold-coated stents did not produce the required outcomes. Dahl et al. reported an increase in neointimal proliferation in individuals receiving stents coated with gold [55]. Danzi et al. observed predominantly proliferative restenosis morphology in 83% of cases and complete occlusion in the remaining 17% [55,56]. These findings indicate that the clinical performance of gold-coated stents did not meet the initial expectations.

### 4.2. Iridium Oxide

Iridium oxide, known for its excellent biocompatibility and inert properties, has been adopted as a stent coating material. Some metals like cobalt, zinc, nickel, copper, silver, chromium, and their alloys can corrode in the body and produce hydrogen peroxide, a potent oxidizing agent that can be detrimental to the artery, triggering inflammation. Coating a metal stent with iridium oxide is believed to facilitate the swift conversion of hydrogen peroxide into harmless water and oxygen [57,58], thereby reducing inflammatory reactions and fostering the formation of a healthy endothelial layer on the stent. Initial studies conducted in a porcine model demonstrated that an iridium-coated stent significantly reduced neointimal thickness compared to a bare stainless-steel stent.

The Lunar stent, developed by Inflow Dynamics, utilizes a base of 316L stainless steel, incorporating a thin internal layer of gold to improve visibility and an external layer of iridium oxide for enhanced biocompatibility [59]. A clinical study investigating the immediate and long-term outcomes of these stents reported an overall angiographic restenosis rate of 13.8% [59]. The presence of the iridium oxide coating was attributed to promoting rapid endothelialization by inhibiting the generation of harmful oxygen radicals, which could otherwise negatively affect the adhesion and growth of endothelial cells. This feature contributes to the improved performance and biocompatibility of the stent in clinical applications. Ongoing research and advancements in stent technology continue to explore innovative ways to optimize stent design and enhance patient outcomes.

### 4.3. Silicon Carbide (SiC)

Silicon carbide (SiC), particularly amorphous hydrogenated SiC, is a semiconductor renowned for its antithrombogenic properties. Its ability to minimize platelet, leukocyte, and monocyte deposition on a stent’s surface makes it a promising surface modifier for addressing restenosis. In vitro studies have shown promising results, indicating the potential of SiC-coated stents in reducing thrombotic events. The findings from various human trials have been contradictory. Some studies reported endothelialization in clinical follow-ups of SiC-coated stents over a 6-month period, suggesting positive outcomes. Conversely, other studies observed increased neointimal hyperplasia within the same follow-up duration for SiC-coated stents, indicating less favorable results [60]. In a clinical trial comparing SiC-coated stents from Biotronik (Hennigsdorf, Germany) with 316L NIR stents from Boston Scientific (Marlborough, Massachusetts, United States of America), no definitive superiority was found between the two stent types in terms of major adverse coronary events during an 8-to-12-week follow-up period [56].

### 4.4. Carbon Coating

Carbon coating, specifically diamond-like carbon (DLC), has been investigated as a surface-modifying material for stents due to its chemical inertness and improved biocompatibility. Certain research has indicated that stents coated with DLC can reduce the occurrences of stent thrombosis and restenosis, particularly in high-risk individuals. An illustrative case is the Carbostent (Sorin Biomedica, Via Crescentino, Saluggia, Italy) study, where a 6-month evaluation revealed a noteworthy reduction in the rate of angiographic restenosis (11%), and there were no indications of subacute thrombosis. The application of DLC-coating on stents appears to hold promise in improving their efficacy and safety profile for patients considered at higher risk for complications following stent implantation. Continued investigations in this area aim to validate and refine these findings, leading to further advancements in stent technology for enhanced patient outcomes [61]. But the results of studies on carbon-coated stents have not been consistent. Some more recent studies have suggested that carbon coatings may be “inactive” in terms of improving angiographic restenosis rates [61]. Clinical studies comparing uncoated stents with carbon-coated stents have shown no significant influence on the inflammatory response or similar rates of binary restenosis between the two types of stents.

## 5. Biocompatible Polymer Stent Coatings

Biocompatible polymers are commonly used as coating materials due to their versatility and ability to be tailored for specific applications. Examples of biocompatible polymers used in stent coatings include polyurethane, polyethylene glycol (PEG), poly (lactic-co-glycolic acid) (PLGA), and poly (vinyl alcohol) (PVA) [6,7]. These polymers can provide a barrier between the stent’s surface and blood components, minimizing platelet adhesion and activation. They can also be modified to incorporate drug-eluting capabilities, enhancing their therapeutic efficacy. When evaluating the polymers applied to coated stents that come into contact with blood, biocompatibility plays a pivotal role. Biocompatibility refers to the response of cells to the presence of a material in their environment. For researchers in the field of polymer science, defining biocompatibility can pose challenges. The polymer used should not elicit an inflammatory reaction and must exhibit the capability to stretch without peeling or separating, especially in the context of drug-eluting stent (DES) applications. Achieving optimal biocompatibility is a critical aspect in the development of medical devices like stents, as it ensures that the materials used interact harmoniously with the body and promote successful treatment outcomes. The assessment of biocompatibility involves rigorous testing and evaluation to ascertain the safety and compatibility of the materials with living tissues, paving the way for the creation of effective and well-tolerated medical interventions.

### 5.1. Biodegradable Polymers for Stents

There are two main categories of polymers used for stent coatings: biodegradable and nonbiodegradable (durable) polymers. Biodegradable polymers are designed to break down gradually over time, reducing the long-term presence of foreign material in the body. They often cause less inflammation and immune response, making them suitable for short-term drug release, typically during the critical early phase after stent placement. This feature can help prevent restenosis while minimizing the risk of late stent thrombosis. In contrast, nonbiodegradable (durable) polymers remain stable within the body, providing long-term support and sustained drug release capabilities. They are suitable for extended drug delivery but may increase the potential for late stent thrombosis and trigger chronic inflammatory responses due to their persistent presence. The choice between these polymers depends on factors such as the clinical scenario, required duration of drug release, and the desired balance between short-term and long-term effects on vessel healing and thrombosis risk.

Researchers have explored various biodegradable polymers for stent coatings, such as polyglycolic acid, polylactic acid, copolymers of these, and poly (ethylene glycol-block-ethylene terephthalate) [6]. On the other hand, for drug-eluting stents (DESs), they have tested durable polymers like methacryloyl phosphorylcholine–laurylmethacrylate, poly(ε-caprolactone), poly (ethylene terephthalate), silicone, and polyurethane [62]. The exact compositions of these polymers are often kept confidential as proprietary information. The investigation of different polymer materials for stent coatings is an ongoing endeavor, seeking to strike a balance between biodegradability and long-term durability while ensuring optimal biocompatibility and therapeutic effectiveness. Despite polyurethanes being categorized as long-lasting polymers, they lack biostability over prolonged durations, and the breakdown products they generate can be harmful. Researchers have explored thermoplastic polyurethanes with shape memory characteristics for DES applications [63]. A hypothesis put forward by Pinchuk suggests that the durability of a polymer in living tissue over time can be compromised if it contains vulnerable groups prone to oxidation, hydrolysis, or enzymatic cleavage, such as ester, amide, ether, carbamate, or urea groups [64]. Polymers containing secondary or tertiary carbon groups, like polyethylene and polypropylene, should be avoided due to the potential for embrittlement and stress cracking caused by double-bond formation. Based on clinical evidence, this hypothesis seems to be supported. Ensuring the long-term safety and effectiveness is of utmost importance, given the debates surrounding the utilization of DESs versus bare metal stents (BMSs), especially in light of late thrombosis cases. The two DESs currently endorsed by the FDA employ poly (ethylene-co-vinyl acetate) (PEVA) and poly (n-butyl methacrylate) (PBMA) (J&J), as well as poly(styrene-b-isobutylene-b-styrene) (SIBS) (BSC) as polymer coatings [65]. The Cypher stent, manufactured by Cordis (J&J), is comprised of 316L stainless steel (low-magnetic, low-carbon) and is coated with a combination of polymers (PEVA: PBMA 67:33) that contains the drug sirolimus [66]. Sirolimus is a natural macrocyclic lactone with immunosuppressive properties, known to inhibit the proliferation of lymphocytes and smooth muscle cells. Clinical studies have exhibited remarkable outcomes with sirolimus-eluting stents, demonstrating reduced restenosis and associated clinical events compared to BMSs.

The Taxus stent, also manufactured with 316L stainless steel, features a polymer known as SIBS (or Translute), combined with paclitaxel (PTx). Paclitaxel, derived from the bark of the Pacific yew tree, has been utilized in cancer treatment. SIBS is a thermoplastic elastomeric biomaterial with phase-separated glassy domains. Extensive clinical trials have showcased the effectiveness and safety of the Taxus stent, leading to its approval by the FDA in 2004. The polymer has demonstrated excellent vascular compatibility, and the drug release kinetics are correlated with the drug-to-polymer ratio [67].

Significant advancements in the field of vascular prostheses have led to the development of polymer-coated stents (PCSs) that offer advantages over traditional metal stents. Polymer coatings provide flexibility and plasticity to facilitate easier placement of the stent at the implantation site. These coatings serve several important functions, including preventing drug wash-off, serving as a scaffold for drug loading, enabling controlled drug release, and ensuring biocompatibility. The top coating layer of the polymer is specifically designed to prevent a burst release of drugs, allowing for a sustained and controlled drug elution at the target site. PCSs face some mechanical limitations such as coating damage, including cracks, flaking, and delamination. The prolonged existence of nondegradable polymers at the site of vessel injury can increase the likelihood of late stent thrombosis (ST). Research findings have led to the establishment of certain requirements for polymer-coated stents (PCSs). First-generation DESs did not meet current medical standards due to concerns about long-term safety, particularly the increased risk of late and very late stent thrombosis. DESs have encountered technical hurdles, including delayed endothelialization due to drugs being delivered locally, the inherent thrombogenicity of the stent as a foreign object, hypersensitivity and inflammatory responses to the stent’s base structure and coatings, insufficient drug dosage, limited sustained drug release, and the potential risk of stent displacement. These challenges have spurred researchers to explore innovative solutions to improve the safety and efficacy of DESs, seeking to address these concerns and enhance patient outcomes.

Polymer-coated stents have significantly advanced the field of interventional cardiology, providing effective solutions for treating coronary artery disease. Ongoing research and innovation continue to refine stent designs, coatings, and materials, with a focus on improving patient outcomes. Researchers are exploring novel approaches to reduce inflammation, enhance drug delivery efficiency, and minimize late stent thrombosis risk. These efforts aim to make stent technology even safer and more effective, offering patients a brighter outlook in the management of cardiovascular conditions.

To address concerns related to inflammation and in-stent restenosis linked to nonbiodegradable polymers, the idea of using biodegradable materials for stent construction has emerged, as shown in Table 3. These second-generation DESs have safer designs with thinner struts, leading to improved biocompatibility and biodegradability. Biodegradable polymers are now utilized as coating materials in DESs to mitigate adverse effects and allow better control over drug release [7].

### 5.2. Bioresorbable Cardiovascular Scaffolds (BRSs)

Bioresorbable cardiovascular scaffolds (BRSs) present a hopeful and viable substitute to permanent stents in the field of cardiology. These innovative scaffolds have the potential to provide temporary support and treatment for diseased blood vessels while gradually degrading within the body over time. Unlike permanent implants, these scaffolds have a temporary nature, gradually degrading over time. Ideal biodegradable scaffolds should possess specific properties, including biocompatibility, sufficient radial strength, controlled degradation within a suitable timeframe (typically 4–6 months), absence of an inflammatory response during degradation, compatibility with drug-eluting technology, thin struts, deliverability convenience, enhanced clarity under fluoroscopy, integration with current delivery systems, and enhanced duration for setup [68]. One significant advantage of biodegradable scaffolds is the absence of a permanent core, overcoming limitations seen in conventional bare-metal stents (BMSs) or metal-based drug-eluting stents (DESs). Once the drug supply is depleted, and the blood vessel has fully healed, the scaffold undergoes a process of bio-reabsorption, enabling the vessel to recover its unobstructed state.

Bioresorbable cardiovascular scaffolds (BRSs) offer a range of benefits, including adaptive shear stress, late luminal gain, reduced restenosis, and late stent thrombosis. Adaptive shear stress denotes the dynamic force of blood flow against the endothelial lining of blood vessels, influencing endothelial cell function and vascular health. Late luminal gain is the increase in blood vessel diameter several months post-stent placement, reflecting the stent’s ability to maintain vessel patency. Reduced restenosis signifies successful inhibition of excessive smooth muscle cell growth, preventing blood vessel re-narrowing following interventions like angioplasty and stent placement. Late stent thrombosis, a concerning complication, refers to blood clot formation within the stent, potentially leading to heart attacks or strokes months or years after implantation. Managing these factors is crucial in enhancing stent efficacy and ensuring optimal patient outcomes. They also have the potential for reintervention at the injury site and improved invasive imaging [68]. BRSs demonstrate superior restoration of unaltered vascular function and elevated flexibility compared to metal-based stents, representing substantial progress in minimally invasive cardiac treatments [7]. Biodegradable scaffolds, while offering potential benefits in medical applications, come with a set of associated risks. One concern is the variability in drug release profiles, which may be less predictable compared to metallic drug-eluting stents (DESs), potentially affecting the effectiveness of the drug in preventing restenosis or thrombosis. Another risk is an increased susceptibility to acute strut fracture when compared to their metallic counterparts. This can compromise the structural integrity of the scaffold, posing potential safety issues. Biodegradable scaffolds may be associated with higher rates of early thrombosis, especially during the degradation process, which raises concerns about their safety. Specific storage and deployment requirements can also be challenging, and the thicker struts of these scaffolds may pose delivery challenges during implantation procedures. Lastly, concerns exist regarding the adequacy of the degradation profiles and the potential for inflammatory degradation residues, which can affect tissue response and long-term outcomes. Scaffold degradation continues to be a concerning issue due to problems with vessel recoil and potential hypersensitivity reactions. Until recently, surgeons typically preferred metal-based stents (BMSs or metal-based DESs) over polymer-based stents because of the superior mechanical strength offered by metal platforms. Heparin loading in metal stents allowed for better control over thrombosis [40]. Metal stents also have their own drawbacks. Despite this, gaining a deeper understanding of the mechanical behavior of polymers during and after implantation and conducting comparative studies between polymer-based and metal-based stents are crucial areas of research. Polymers are favored for their versatility, yet their mechanical properties profoundly influence their performance and long-term success. During implantation, these materials encounter various mechanical stresses, including compression, expansion, bending, and torsion, depending on the device and its location within the body. Designing polymers capable of withstanding these forces is essential. Biomechanical compatibility is also crucial. The mechanical properties of polymers must harmonize with those of surrounding biological tissues to prevent complications like stress concentration, which could lead to implant failure or tissue damage. Potential complications associated with polymer-based stents include lower stiffness and strength compared to conventional metal-based stents, an increased strut diameter leading to complications such as platelet adhesion and vessel injury, and premature polymer destruction at stress levels below its yield and tensile strength [7,8,68].

Completely bioresorbable coronary scaffolds are medical instruments crafted from polymeric substances, offering transient reinforcement to the blood vessel at the targeted treatment area and delivering medications locally. These scaffolds gradually resorb and dissolve within the body over a period of several months to years. Unlike traditional metallic stents, which remain permanently in the treated vessel, bioresorbable scaffolds have the advantage of avoiding permanent caging of the stented segment. The use of bioresorbable scaffolds holds the potential to offer various advantages, surpassing the existing metallic stent platforms. By allowing the treated vessel to uncage over time, these scaffolds might enhance the durability of the vessel over time, maintain physiologic vasomotion (the ability of the vessel to contract and relax), adapt to changes in shear stress, and promote the sealing of atherosclerotic plaques. Evidence suggests that after the implantation of bioresorbable scaffolds, the treated atherosclerotic plaque can be sealed by the formation of a neocap, and vessel remodeling featuring lumen expansion and mitigation of plaque build-up can be observed in both animal models and humans. The summaries of different types of bioresorbable coronary scaffolds are shown in Table 4.

### 5.3. Nonbiodegradable Stents

Extensive research has been conducted on a wide range of polymers to explore their potential application in stent technology. Synthetic polymers offer advantages such as biocompatibility, predictable properties, and consistent performance across different batches, making them preferable to natural polymers. When selecting a polymer for a drug delivery system, factors like biodegradability and biocompatibility are crucial considerations. The selected biodegradable polymer must undergo efficient metabolism and elimination from the body, breaking down into non-toxic byproducts and avoiding any inflammatory responses. The initial drug-eluting stents, such as Cypher^®^ and Taxus^®^, employed a stainless-steel structure coated with nonbiodegradable polymers like PBMA, PEVA, or PSIBS. These stents were effective in reducing restenosis but raised concerns about late stent thrombosis with long-term use.

Addressing these concerns, the next-generation drug-eluting stents introduced thinner struts and integrated novel and more potent drugs. One of the most significant improvements is the adoption of biodegradable polymers for the stent’s coating. These polymers gradually break down over time, reducing the risk of long-term inflammation and vessel damage that can occur with nonbiodegradable alternatives. These advanced DESs feature thinner stent struts, enhancing stent flexibility and minimizing the potential for vessel injury during deployment. Thinner struts also contribute to a decreased risk of restenosis, as they allow for more natural vessel healing. Next-generation DESs incorporate more potent and targeted drug formulations, ensuring a more efficient inhibition of cell proliferation. This enables the use of lower drug doses, reducing potential side effects while maintaining effective treatment. To enhance stent performance and reduce complications associated with nonbiodegradable polymers, synthetic nonbiodegradable polymers such as PBMA, PEVA, PSIBS, PHFP, and PVDF were combined with phosphorylcholine polymer (PCh) [69]. The Cypher^®^ stent, produced by Cordis Corporation, consists of a stainless-steel (SS) platform coated with a polymer system comprising poly (ethylene-co-vinyl acetate) (PEVA), poly (n-butyl methyl acrylate) (PBMA), and phosphorylcholine polymer (PCh) [66]. It releases sirolimus, with drug release percentages of 40% at 5 days, 85% at 30 days, and 100% at 90 days. The Cypher^®^ stent has received approval from both the FDA and CE. Another well-known stent is the Taxus^®^ stent, developed by Boston Scientific, which employs a stainless-steel platform and is coated with poly(styrene-b-isobutylene-b-styrene) as the polymer system. It releases paclitaxel, with a drug release percentage of less than 10% at 28 days. The Taxus^®^ stent is also FDA- and CE-approved [67].

Several stents from different manufacturers have received Food and Drug Administration (FDA) and Conformité Européene (CE) approval. The Promus PREMIERTM stent by Boston Scientific has a platinum–chromium platform coated with poly (n-butyl methyl acrylate) and poly(vinylidene-co-hexafluoropropylene) as the polymer system, releasing everolimus over 28 days (71%) and 120 days (100%) [70]. The Xience V^®^ stent by Abbot Vascular features a cobalt–chromium platform with a similar polymer system, releasing everolimus over 28 days (80%) and 120 days (100%) [71]. The Firebird 2^®^ stent by Essen Technology in Beijing features a cobalt–chromium platform coated with poly (styrene-butylene styrene) and releases sirolimus over 7 days (50%) and 30 days (90%) [72].

The latest breakthrough in less invasive treatment for coronary artery disease (CAD) revolves around the emergence of fourth-generation DESs. These stents integrate a biodegradable polymer core combined with an active substance. Essential components like biodegradable polymers such as poly (lactic acid) (PLA), poly (glycolic acid) (PGA), poly (lactic-co-glycolic acid) (PLGA), and poly(caprolactone) (PCL) are pivotal in the stent’s design, providing favorable characteristics like degradability, biocompatibility, and mechanical robustness. These polymers have been effectively utilized in various medical applications, including drug delivery systems, implants, sutures, and stent scaffolds. PLA is widely used in medical applications, being biodegradable, bioresorbable, and FDA-approved. PGA has excellent mechanical properties but may cause inflammation due to acidic byproducts [73]. PLGA, a copolymer of PLA and PGA, offers tunable degradation rates. PCL is a biocompatible, low-cost polymer with long degradation times due to its relatively stable ester linkages, higher molecular weight options that have more ester bonds to break, its hydrophobic nature which slows down water penetration and hydrolysis, and its crystalline structure that can impede the degradation process, collectively contributing to its extended degradation timeline in biomedical applications. Polyurethanes (PUs) are also gaining attention for their biomedical applications, as they can be engineered to be biocompatible, adaptable to specific needs, and mimic the mechanical properties of natural tissues, making them suitable for a wide range of medical devices and implants [63].

## 6. Biomimetic Coatings

A biomimetic coating on a stent refers to a specialized surface treatment or layer that is designed to mimic or imitate the natural biological environment within the human body. These coatings often consist of bioactive peptides or proteins that can enhance cell adhesion, proliferation, and migration. Examples include coatings incorporating peptides derived from fibronectin or collagen, which are important components of the ECM. Fibronectin and collagen are essential components of the ECM because they play pivotal roles in cell adhesion, proliferation, and tissue development. Fibronectin, for instance, acts as a bridge between cells and the ECM, facilitating cell attachment and migration, while collagen provides structural support and influences cell behavior, making them integral to tissue regeneration and repair processes. Biomimetic coatings provide a supportive environment for endothelialization, contributing to improved stent hemocompatibility. The development of effective re-endothelialization of procedures for intravascular implants is critical for preventing thrombosis and ensuring biocompatibility. Re-endothelialization is the process of repairing and regenerating the endothelial layer that lines the inner surface of blood vessels, playing a crucial role in maintaining vascular health by regulating blood flow and preventing clot formation. Inadequate re-endothelialization can lead to complications such as restenosis, thrombosis, inflammation, and impaired vasomotor function, emphasizing the importance of supporting this regenerative process in medical interventions and treatments to avoid adverse vascular outcomes. One approach is the use of biomimetic coatings composed of an endothelial extracellular matrix (EC-ECM) and specific micropatterns [74]. These coatings have shown good blood compatibility, anti-inflammatory properties, and the ability to promote endothelialization while inhibiting smooth muscle cell proliferation and macrophage attachment. Researchers have successfully created biomimetic coatings by culturing endothelial cells (ECs) on micropatterned surfaces and then decellularizing the resulting ECM. By incorporating hyaluronic acid (HA) micropatterns, cell morphology is prolonged, leading to increased release of anticoagulant factors and limiting the contractile phenotype of smooth muscle cells (SMCs) [74]. This approach improves the biocompatibility of the EC-ECM coating, as demonstrated by its anticoagulation, endothelialization, and anti-inflammatory properties. To enhance the functionality of the coating, researchers have explored the creation of composite coatings by incorporating both SMC-ECM and EC-ECM. Imitating the natural vascular basement membrane, they cultured SMC and EC sequentially on polydopamine-coated surfaces and then decellularized the resulting ECM. It reduces rupture or destruction of red blood cell (erythrocytes) hemolysis on the material surface.

To facilitate the application of ECM coatings on biodegradable or uneven materials, researchers have developed methods of dispersing the ECM into a solution and self-assembling it onto the material surface [74]. Heparin, a natural polysaccharide with antithrombotic properties, can be immobilized on the ECM coating to selectively promote EC proliferation while inhibiting SMC growth, further enhancing blood compatibility [40]. The molecular weight (MW) of hyaluronic acid has an important role in the biocompatibility of the coatings. High MW HA inhibits the adhesion of platelets, SMC, and macrophages, providing anticoagulant, anti-proliferative, anti-inflammatory, and non-immunogenic properties [74]. Extremely high MW HA may hinder endothelialization. Researchers have successfully prepared coatings with HA of gradient MW, allowing for better versatility and control over the surface properties. The use of HA nanoparticles carrying magnesium (Mg) ions has been explored [75]. Mg ions can inhibit EC apoptosis and promote nitric oxide release, contributing to endothelial cell function and preventing thrombosis. In the case of biodegradable Mg alloys, which degrade to release Mg ions, a nanocomposite coating can be used to regulate Mg transportation to EC, SMC, and macrophages based on their specific requirements [76].

**Figure 6:**
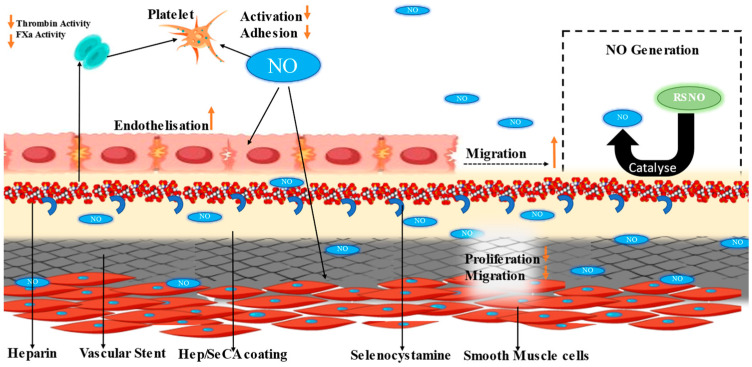
Endothelium-mimetic coating for cardiovascular stents by combining heparin and nitric oxide (NO). Heparin/selenocystamine (Hep/Se CA), factor Xa (FXa), nitric oxide (NO), S-nitrosothiols (RSNO).

In research conducted by [47], as can be seen in Figure 6, researchers developed an endothelium-mimetic coating for cardiovascular stents by combining heparin and nitric oxide (NO). The coating prevents thrombosis, supports endothelial cell growth, and suppresses smooth muscle cell proliferation. The surface promotes a contractile phenotype in smooth muscle cells while creating a favorable microenvironment for endothelial cells. This coating significantly improves stent antithrombogenicity, re-endothelialization, and anti-restenosis in vivo.

## 7. Surface Modification Techniques

Surface modification techniques for stents refer to a set of processes and methods used to alter or enhance the outer surface properties of these medical devices. These techniques are applied to improve the stent’s biocompatibility, functionality, and overall performance.

Multiple surface modification techniques can be utilized to improve stent hemocompatibility. Among them are plasma treatment, physical vapor deposition (PVD), chemical vapor deposition (CVD), and electrodeposition [77]. These methods enable precise manipulation of surface properties, including roughness, charge, and composition, to diminish platelet adhesion, modify wettability, or facilitate the release of bioactive agents. Surface modification techniques can be combined with coating materials to optimize the desired hemocompatible properties.

### 7.1. Plasma Oxidation

Plasma oxidation is a surface modification technique used to enhance stent hemocompatibility by improving the interaction between the stent’s surface and blood components. During the process, the stent’s surface is exposed to ionized gas (plasma), which creates chemical functional groups on the surface, making it more hydrophilic and negatively charged. This modification reduces the stent’s thrombogenicity, preventing blood clot formation, and promotes better blood compatibility by reducing platelet adhesion and activation. Enhanced surface wettability facilitates the formation of a stable and uniform endothelial cell layer, further contributing to improved hemocompatibility and reducing the risk of adverse events such as thrombosis or restenosis. It also involves generating oxide layers on the surface through the transfer of energy from plasma. This process increases the surface energy of the material, making it more reactive and capable of forming nanocrystalline stoichiometric TiO_2_ oxide layers [78]. Studies have shown that having a rutile TiO_2_ phase on the surface can decrease thrombosis. Plasma oxidation has been shown to augment the roughness of the oxide layer, influencing the hydrophilicity of the surface. Chiang et al. conducted research on plasma-oxidized titanium surfaces and observed that samples with a rugged dimple-like oxide layer and a nanostructured rutile TiO_2_ phase exhibited improved compatibility with blood (hemocompatibility) [79]. They subjected pure titanium surfaces to oxygen plasma at different treatment powers and durations to generate a titanium oxide layer. Microscopic analysis unveiled the formation of island-like and dimple-like nanostructured rutile TiO_2_ layers on the plasma-oxidized titanium surface. These findings contribute to advancing our understanding of surface modifications, offering potential benefits for enhancing the biocompatibility of biomaterials used in medical devices and implants. Continuous exploration of plasma oxidation techniques opens avenues for optimizing surface properties and ultimately improving patient outcomes. The existence of a rough dimple-like oxide layer with nanostructured rutile TiO_2_ indicated enhanced hemocompatibility compared to control surfaces.

### 7.2. Physical Vapor Deposition (PVD)

Physical vapor deposition (PVD) technology finds widespread application in applying coatings on medical devices, including orthopedic devices and cardiac stents. PVD allows for the deposition of various coating materials, such as TiN (titanium nitride), titanium carbon nitride (TiCN), chromium nitride (CrN), titanium aluminum nitride (TiAlN), and diamond-like carbon (DLC) on these devices [78]. These coatings modify the surface properties of the devices while preserving their biomechanical properties. In the context of coronary stents, one example of a coating material applied using PVD technology is TiN. TiN coatings offer excellent biocompatibility and corrosion resistance, making them suitable for cardiovascular implants. These coatings can enhance the hemocompatibility of stents, reduce platelet adhesion, and promote endothelialization, thereby improving their performance and long-term functionality [79]. Other coating materials used for coronary stents may include TiCN, CrN, TiAlN, and DLC. These coatings offer various advantages such as improved mechanical properties, reduced friction, enhanced wear resistance, and minimized restenosis.

### 7.3. Chemical Vapor Deposition (CVD)

Chemical vapor deposition (CVD) is a deposition method where gases chemically react with a substrate, leading to the creation of a nonvolatile compound on the surface. Unlike physical vapor deposition (PVD), which relies on physical processes like evaporation and sputtering, CVD utilizes chemical reactions to deposit thin films [80]. Various chemical vapor deposition (CVD) techniques have been developed, such as atmospheric-pressure (APCVD), low-pressure (LPCVD), plasma-enhanced (PECVD) or plasma-assisted (PACVD), and laser-enhanced (LECVD) techniques. Chemical vapor deposition (CVD) encompasses several techniques, each suited to particular applications. Atmospheric-pressure CVD (APCVD) is conducted at room pressure, making it cost-effective, and is often used for large-area coatings, such as architectural glass and flat-panel displays. Low-pressure CVD (LPCVD) operates under reduced pressure, offering precise control and high-purity film growth, commonly employed in semiconductor manufacturing. Plasma-enhanced CVD (PECVD) uses plasma activation to enhance chemical reactions, finding extensive use in microelectronics, photovoltaics, and protective coatings. Laser-enhanced CVD (LECVD) combines lasers with CVD processes for precise film deposition in applications like micro-optics and sensor manufacturing. CVD is widely employed in the industry to deposit both organic and inorganic films on various materials, including metals, semiconductors, and more. The process involves several sequential steps, including the transportation of reactants to the reaction zone, chemical reactions in the gas phase, adsorption and diffusion of species onto the substrate surface, heterogeneous reactions leading to film formation, desorption of byproducts, and transportation of reaction byproducts away from the reaction zone. CVD has been utilized to deposit biocompatible thin films like diamond-like carbon (DLC) and diamond films. DLC is a robust and corrosion-resistant material that contains a substantial fraction of sp3 bonds. It has demonstrated excellent biocompatibility in orthopedic and cardiovascular applications. Studies have shown that DLC coatings on stents exhibit reduced cell adhesion and activation compared to traditional materials like titanium (Ti).

### 7.4. Electrodeposition

The electrodeposition technique is a highly effective method for the surface coating of stents, offering precise control over coating thickness, composition, and adhesion. To begin the process, the stent’s surface is meticulously prepared through cleaning and pre-treatment to ensure it is free from contaminants and conducive to adhesion. The stent is immersed in an electrolyte solution containing metal ions or alloy precursors tailored to meet specific coating requirements. In an electrochemical setup, an electric current is applied between the stent (working electrode) and a counter electrode, driving the reduction in metal cations from the electrolyte onto the stent’s surface. This results in the formation of a metal or alloy coating layer whose properties can be fine-tuned by adjusting parameters like voltage, current density, and deposition time. Rigorous quality control measures, including microscopy and corrosion testing, are employed to assess coating integrity. For medical stents, biocompatible materials may be incorporated into the coating, ensuring compatibility with bodily tissues and fluids.

## 8. Evaluation Methods for Hemocompatibility and Thrombogenicity

Reliable evaluation techniques are paramount for the development and optimization of bioactive coatings for several crucial reasons. They allow researchers to accurately assess the hemocompatibility of these coatings by studying their interaction with blood components like platelets and clotting factors. Understanding the hemocompatibility profile helps identify potential thrombogenic risks, enabling researchers to optimize coatings to minimize thrombus formation and enhance the safety of stent implantation. These evaluation techniques provide valuable insights into the clinical performance of bioactive coatings. By closely mimicking physiological conditions in in vitro assays and animal models, researchers can simulate relevant biological processes and assess factors such as platelet adhesion, coagulation, endothelialization, and inflammatory response. This predictive information aids in determining the long-term efficacy, potential adverse events, and overall performance of coatings in real clinical settings. Reliable evaluation techniques facilitate direct comparisons between different coating formulations. By using standardized methods, researchers can objectively evaluate the performance of various coatings and identify the most promising candidates for further development. Such techniques are essential for obtaining regulatory approval for clinical use. By demonstrating the safety and efficacy of bioactive coatings through robust evaluation techniques, researchers ensure that these coatings can be safely and effectively used in patients.

Assessing the hemocompatibility and thrombogenicity of stent coatings requires a comprehensive evaluation using both in vitro and in vivo methods. Some of the evaluation methods commonly employed are:

1. In vitro platelet adhesion and activation assays: Platelet adhesion and activation assays involve exposing stent coatings to platelet-rich plasma or whole blood in controlled laboratory settings. These assays measure the extent of platelet adhesion and activation on the coated surface using techniques such as scanning electron microscopy (SEM), flow cytometry, or immunofluorescence staining. By quantifying platelet attachment and activation markers, these assays provide valuable insights into the thrombogenic potential of stent coatings [81].
2. Coagulation assays: Various coagulation assays can be used to assess the impact of stent coatings on the clotting cascade. Prothrombin time (PT) and activated partial thromboplastin time (aPTT) tests measure clotting time in the presence of the coating to evaluate the intrinsic and extrinsic coagulation pathways, respectively. Prothrombin time (PT) and activated partial thromboplastin time (aPTT) are critical blood tests that provide insights into distinct aspects of the coagulation or clotting process within the bloodstream. PT measures the time it takes for blood to clot through the extrinsic and common coagulation pathways, assessing factors like fibrinogen, prothrombin, and factors V, VII, and X. It is particularly useful for monitoring anticoagulant therapy, such as warfarin, and diagnosing conditions like liver disease and clotting disorders. On the other hand, aPTT assesses the clotting time via the intrinsic and common pathways, focusing on factors like VIII, IX, XI, and XII. It aids in diagnosing clotting disorders like hemophilia and monitoring heparin therapy. Thrombin generation assays can assess the effect of coatings on thrombin activity, while fibrinogen adsorption assays provide insights into the coating’s interaction with fibrinogen and its potential to initiate clot formation [82].
3. Platelet function tests: Platelet function tests evaluate the functional response of platelets to stent coatings. Aggregometry measures the ability of platelets to aggregate when exposed to coating surfaces, indicating platelet activation and aggregation potential. Flow-based assays, such as microfluidic systems or perfusion chambers, mimic blood flow conditions and assess platelet adhesion, aggregation, and thrombus formation on stent coatings under shear stress conditions [83].
4. Endothelial cell studies: Evaluating the interaction between stent coatings and endothelial cells is essential for assessing their hemocompatibility. Endothelial cell adhesion, proliferation, and morphology can be analyzed using techniques like cell viability assays, immunostaining, or scanning electron microscopy. In vitro studies can provide insights into the coating’s ability to promote endothelialization and prevent thrombus formation [84].
5. Animal models and in vivo studies: Animal models are critical for assessing the hemocompatibility and thrombogenicity of stent coatings in a physiological context. Implanting coated stents in animal models allows for the evaluation of factors such as thrombus formation, neointimal hyperplasia (refers to the abnormal and excessive proliferation or growth of smooth muscle cells within the innermost layer of an artery, known as the intima), endothelialization, and inflammatory response. Various animal models, such as rats, rabbits, or pigs, are used to simulate human vascular environments and assess the safety and efficacy of stent coatings [85].

Continued advancements in these assay techniques and models enable researchers to refine and optimize stent coatings, improving their ability to minimize thrombus formation and enhance patient safety during cardiovascular interventions.

## 9. Recent Advances

Recent advancements in the surface engineering of bioactive coatings for stents have been focused on enhancing their hemocompatibility, reducing thrombogenicity, and improving long-term performance.

Stents coated with long-lasting polymers may lead to delayed healing of the arterial wall. To mitigate the risk of stent thrombosis, researchers have introduced biodegradable polymer coatings such as Biomatrix, Nobori, and Yukon ChoicePC. Biomatrix is known for its innovative biodegradable polymer coating, which gradually releases an anti-proliferative drug (typically sirolimus) to inhibit smooth muscle cell growth and reduce the risk of restenosis. Nobori, on the other hand, features a similar design with a biodegradable polymer but utilizes a different drug, biolimus A9, to achieve the same goal of preventing cell proliferation within the artery. Both Biomatrix and Nobori aim to minimize long-term complications associated with durable polymer DESs. In contrast, Yukon ChoicePC offers versatility with options for both bare-metal and drug-eluting stents. Extensive clinical trials have demonstrated comparable outcomes with the biodegradable polymer-based Nobori biolimus-eluting stents [86]. The Synergy stent, built with a 74 mm thick platinum–chromium platform, releases everolimus through a 4 mm thick PLGA polymer coating. In a randomized evaluation of the XIENCE V Everolimus Eluting Coronary Stent System, Phase I Clinical Investigation (EVOLVE I) non-inferiority trial, 291 patients received a full dose, half dose, or Promus element stents. Both platforms exhibited similar clinical results and demonstrated non-inferiority to the Promus element stent at the 6-month mark. The Synergy full dose platform has obtained CE approval and is presently undergoing evaluation in the more extensive evaluation of the XIENCE V Everolimus Eluting Coronary Stent System, Phase II Clinical Investigation (EVOLVE II trial) [87].

The development of biodegradable polymer coatings in stent design aims to enhance arterial healing and reduce long-term complications associated with permanent polymers. Biodegradable coatings allow for a gradual release of the drug to promote a controlled healing response without causing undue inflammation or delayed endothelialization. The Synergy stent’s platinum–chromium platform and PLGA polymer coating have been engineered to optimize drug release and ensure effective treatment over the desired period. The ongoing EVOLVE II trial seeks to validate and further refine the clinical performance of the Synergy stent, bringing us one step closer to safer and more efficacious interventional cardiology interventions.

The Orsiro stent, featuring a 60 mm thick cobalt–chromium structure, delivers sirolimus from a biodegradable polymer coating and includes a silicon carbide coating to minimize corrosion. The BioFLOW-II trial demonstrated no inferiority in in-stent LLL (late lumen loss) at 9 months, with comparable in-segment binary restenosis between Orsiro and Xience Prime. Numerous ongoing trials are currently comparing the Orsiro stent with other newer-generation drug-eluting stents (DESs) [88].

The DESyne BD stent, an 81 mm thick cobalt–chromium stent, integrates a biodegradable polymer coating. It has shown non-inferiority to the Endeavor ZES for in-stent LLL at 6 months and significantly reduces angiographic binary restenosis compared to the Endeavor ZES [89]. In-stent late lumen loss (LLL) and angiographic binary restenosis are two critical concepts in the evaluation of the effectiveness of stents used in coronary artery interventions. In-stent LLL refers to the measurement of the reduction in the inner diameter of a stented coronary artery at a certain time after the stent placement, typically measured in millimeters. This measurement helps assess the degree of re-narrowing or re-blockage of the treated artery over time, with lower LLL values indicating better long-term outcomes.

Angiographic binary restenosis, on the other hand, is a dichotomous assessment that determines whether there is significant re-narrowing (restenosis) of the stented artery or not. It involves comparing the post-procedure angiogram (X-ray image) with a follow-up angiogram to determine if there is a significant reduction in the diameter of the treated artery. If the artery’s diameter has decreased beyond a certain threshold, usually 50% or more, it is considered angiographic binary restenosis, signifying a potential need for further intervention to reopen the artery.

The Orsiro and DESyne BD stents represent significant advancements in stent technology, aiming to improve long-term clinical outcomes for patients. The incorporation of biodegradable polymer coatings in these stents addresses concerns related to the long-term presence of durable polymers, potentially reducing the risk of delayed healing and inflammation. The use of cobalt–chromium as a stent material enhances the mechanical properties and structural integrity, contributing to improved stent performance. Ongoing comparative trials with other DESs aim to establish the relative efficacy and safety profiles of these newer-generation stents, further advancing the field of interventional cardiology and enhancing treatment options for patients with coronary artery disease.

The combo stent utilizes a robust stainless-steel scaffold measuring 100 mm, coated with a biodegradable polymer and an anti-CD34 antibody coating. The CD34 antibody is a monoclonal antibody with applications in various medical and research fields, and it primarily targets the CD34 antigen found on the surfaces of certain cells, including hematopoietic stem cells and endothelial progenitor cells. Anti-CD34 antibody coatings on stents offer several noteworthy advantages. Firstly, they promote endothelialization, facilitating the adhesion and proliferation of endothelial progenitor cells on the stent’s surface. This process, known as re-endothelialization, results in the formation of a healthy endothelial cell layer, reducing the risk of complications such as thrombosis and restenosis. Research indicates that this combination enhances endothelialization, reduces neointimal hyperplasia, and lowers inflammation when compared to standard sirolimus-eluting stents (SESs) and stents coated solely with anti-CD34 antibodies [90]. The unique design of the combo stent aims to optimize the healing response and improve long-term outcomes for patients undergoing coronary interventions.

The BioFreedom polymer-free biolimus-eluting stent, composed of stainless steel, underwent evaluation in a first-in-man study. The stent demonstrated superior angiographic end points of in-stent late lumen loss (LLL) at 4 and 12 months, with both standard and low doses of BioFreedom showing superiority over other stents. In addition, the study compared BioFreedom with bare-metal stents (BMSs) in 2456 patients at high risk of bleeding [91]. The BioFreedom stent represents a promising alternative to traditional drug-eluting stents, offering the advantage of a polymer-free design and the controlled release of the drug biolimus to promote arterial healing while minimizing the risk of inflammation and delayed endothelialization.

Another notable area of progress involves the development of multifunctional coatings that combine various functionalities to enhance stent performance. These coatings may incorporate nanostructures, anticoagulant agents, or growth factors to simultaneously reduce platelet activation, inhibit thrombus formation, promote endothelialization, and prevent restenosis. By integrating multiple features into a single coating, multifunctional coatings aim to improve the overall biocompatibility and efficacy of stents.

Nanotechnology has also made significant contributions to surface engineering. Nanostructured coatings, such as nanotubes or nanopatterned surfaces, offer precise control over surface properties, including topography, roughness, and surface energy. These nanoscale features can influence cellular behavior, reduce platelet adhesion, promote endothelial cell growth, and improve drug release kinetics. The research conducted by Vishnu et al., 2020, investigated the impact of hydrothermally treated beta-type Ti single-bond 29Nb alloy on nanostructured titanium surfaces [92]. Successful fabrication of nanograss-like structures with nanotopographies and anatase titania has been achieved. These nanograss structures exhibit superhydrophilic properties, leading to reduced hemolysis rates and minimal platelet adhesion and activation. The development of such superhydrophilic surface coatings opens up new possibilities for blood-contacting implant applications.

A study conducted by Park et al. revealed that while the restoration of damaged endothelium in stent treatment for vascular diseases shows promise, the current outcomes are still insufficient due to recurrence rates [93]. To address this, a novel stent was designed, incorporating anti-CD146 antibody immobilized silicone nanofilaments (SiNf) to more efficiently and specifically capture late endothelial progenitor cells (EPCs). Anti-CD146 antibodies are versatile tools used in both research and clinical applications. These monoclonal antibodies target the CD146 antigen, also known as MCAM or Muc18, which is found on the surface of various cell types, including endothelial cells, melanoma cells, immune cells, and pericytes. In angiogenesis research, anti-CD146 antibodies play a crucial role by helping researchers’ study CD146’s involvement in vascular development and angiogenesis, where new blood vessels form from existing ones. The modified substrates demonstrated the capture of eight times later EPCs and three times more mesenchymal stem cells compared to unmodified ones. The CD146 Ab-armed nanofilamentous stent exhibited excellent performance in reducing thrombosis and restenosis through enhanced re-endothelialization. Stainless-steel coronary stents face in-stent restenosis risks, impacting long-term safety and efficacy. The work by Mohan and co-researchers was aimed at developing a drug-free, polymer-less surface using titania nanotexturing through hydrothermal processing [94]. The nanotextured coatings offered mechanical stability and corrosion resistance, and in vitro studies show faster endothelialization and reduced smooth muscle cell proliferation. This stable, scalable strategy could be a cost-effective alternative to drug-eluting stents for in-stent restenosis.

Researchers are investigating the use of nanoparticles loaded with therapeutic agents, such as drugs or growth factors, for targeted and controlled drug delivery from the coating. Bioactive molecules and peptides derived from the extracellular matrix (ECM) have emerged as promising components to enhance stent coatings. Molecules like RGD peptides can promote cell adhesion, migration, and proliferation, facilitating endothelialization and reducing thrombogenicity. By mimicking the natural environment, these bioactive molecules aid in regeneration and healing processes, further improving biocompatibility. Surface modifications continue to be refined and optimized. Heparin coatings provide localized anticoagulant effects, inhibiting clotting factors and platelet activation. Surface modifications that promote endothelialization aim to create a protective endothelial cell layer, reducing platelet adhesion and activation. Anti-fouling coatings prevent the non-specific binding of proteins and cells, thereby reducing the risk of thrombus formation [47]. In parallel with advancements in surface engineering, evaluation techniques for bioactive coatings have also seen progress. Advanced in vitro models, such as microfluidic systems and biomimetic platforms, enable more accurate and physiological assessment of coating performance. Further improvements in imaging techniques, such as high-resolution microscopy and molecular imaging, allow researchers to visualize and analyze coating–cell interactions at the nanoscale level, providing valuable insights into the mechanisms underlying hemocompatibility and thrombogenicity. These recent advancements collectively contribute to the development of bioactive coatings that are safer, more effective, and better suited for stent applications. As research in this field continues, the potential for further innovations in surface engineering and evaluation techniques holds great promise for improving patient outcomes and reducing complications associated with stent implantation.

## 10. Conclusions and Future Prospects

The future prospects of surface engineering for improved stent hemocompatibility are highly promising and hold great potential for advancing cardiovascular treatments. Continued advancements in nanotechnology will allow for precise control over coating properties, enabling the development of nanostructured coatings and nanoparticles that enhance biocompatibility and reduce thrombogenicity. Personalized medicine may lead to the creation of stent coatings tailored to individual patient needs, optimizing treatment outcomes. Biomimetic coatings, utilizing bioactive molecules and peptides from natural sources, offer the potential for better integration with the body and improved endothelialization. Researchers will focus on refining drug delivery mechanisms to achieve controlled and targeted drug release profiles, enhancing therapeutic efficacy while minimizing side effects. Biodegradable coatings that gradually dissolve after serving their purpose could reduce long-term inflammation and improve outcomes. Advancements in evaluation techniques, such as advanced in vitro models and high-resolution imaging, will provide deeper insights into coating–cell interactions and biocompatibility. Multi-modal approaches, combining drug-eluting capabilities with growth factor promotion and other functionalities, could lead to synergistic effects, further enhancing stent performance. Ongoing research in these areas is poised to significantly enhance the safety and effectiveness of stent implantation, ultimately leading to improved cardiovascular treatments and better patient outcomes.

In conclusion, the discussion on surface engineering for stent coatings highlights several crucial advancements that have the potential to revolutionize medical treatments and improve patient outcomes. Nanotechnology-based coatings offer enhanced properties, making them suitable for various applications, while bioactive coatings with controlled drug release systems hold the promise of providing targeted therapeutic effects and promoting tissue regeneration. The future of surface engineering for stent coatings lies in developing biocompatible and bioactive coatings that mimic the extracellular matrix, incorporate bioactive molecules, and facilitate cell adhesion and growth. To fully realize the potential of drug-eluting coatings for stents, further research is needed to improve drug delivery efficiency, achieve controlled release profiles, and minimize side effects. By enhancing stent hemocompatibility and reducing thrombogenicity, surface-engineered bioactive coatings have the potential to significantly improve patient outcomes in the treatment of cardiovascular conditions. Looking ahead, future research should focus on optimizing coating materials, refining drug delivery mechanisms, and conducting thorough biocompatibility and safety assessments to ensure the efficacy and safety of surface-engineered bioactive coatings. Such coatings also hold promise beyond stents and can potentially be applied to other medical devices and implants, enhancing their performance and biocompatibility in various clinical settings.

## Figures and Tables

**Table 1:** Clotting factors involved in blood coagulation [12,13].

Clotting Factor	Clotting Factor Name	Function	Plasma Half-Life (Hours)	Plasma Concentration (mg/L)
Factor I	Fibrinogen	Converts to fibrin, forms clot mesh	4	1.5–4.0
Factor II	Prothrombin	Converts to thrombin, activates clotting cascade	60–72	0.12–0.16
Factor III	Tissue factor	Initiates extrinsic pathway of coagulation	N/A	Trace amounts
Factor IV	Calcium	Cofactor in multiple coagulation reactions	N/A	2.2–2.7
Factor V	Labile factor	Cofactor in prothrombinase complex	12–18	8–20
Factor VII	Stable factor	Initiates extrinsic pathway of coagulation	2–6	0.5–2.0
Factor VIII	Antihemophilic factor A	Cofactor in intrinsic pathway, enhances factor IX	8–12	0.02–0.20
Factor IX	Christmas factor	Activates factor X in intrinsic pathway	18–24	0.1–0.2
Factor X	Stuart–Prower factor	Prothrombin is transformed into thrombin	24–48	5–15
Factor XI	Plasma thromboplastin antecedent (PTA)	In the intrinsic pathway, it triggers the activation of factor IX	40–60	0.05–0.15
Factor XII	Hageman factor	It initiates the coagulation intrinsic pathway	48–72	0.03–0.08
Factor XIII	Fibrin-stabilizing factor	It forms cross-links within fibrin, providing stability to the clot	10–14	0.02–0.05

**Table 2:** The factors and mechanisms contributing to thrombosis on the surface of stents.

Factor Leading to Thrombosis of Stent Surface	Mechanism	Prevention Measures	Ref.
Activation of Platelets	- Exposure of adhesive proteins. - Disruption of endothelial cell signaling. - Altered blood flow patterns.	- Antiplatelet therapy. - Surface modifications to reduce platelet activation.	[16]
Inflammation and Endothelial Dysfunction	- Local inflammation. - Impaired endothelial cell function. - Release of procoagulant factors.	- Anti-inflammatory medications - Optimal stent sizing and deployment.	[17]
Disruption of Blood Flow	- Disturbed or turbulent flow patterns.	- Stent design optimization to minimize flow disturbance	[18]
Stent Surface Characteristics	- Surface roughness, charge, composition. - Increased platelet adherence and activation.	- Surface coatings to reduce thrombogenicity. - Materials with lower thrombotic potential.	[19]
Delayed Endothelialization	- Incomplete or delayed regrowth of endothelial cells.	- Drug therapies promoting endothelialization. - Stent designs promoting endothelial coverage.	[20]
Drug-Eluting Stents	- Effects of released drugs on endothelial cell function and endothelialization.	- Optimization of drug release kinetics. - Balancing anti-restenosis and antithrombosis effects.	[21]
Stent Under-expansion or Malposition	- Gaps or uneven surfaces promoting platelet adhesion.	- Optimal stent sizing and deployment. - Intravascular imaging guidance.	[22]

**Table 3:** Biodegradable polymer coatings in drug-eluting stents.

Stent	Manufacturer	Polymer Material	Biodegradation	Coating Location and Thickness	Drug Release
Synergy	Boston Scientific	PLGA	3–4 months	Located on the outer surface, at a distance of 4 μm.	50% for 30 days
Orsiro	Biotronik	PLLA	1–2 years	Spread evenly around the circumference, spanning 7 μm.	50% for 30 days
DESyne BD	Elixir Medical	PDLLA	6–9 months	Evenly distributed around the circumference, measuring less than 3 μm.	90% for 90 days
Combo	OrbusNeich	PDLLA, PLGA	3 months	Situated on the outer side, extending to a distance of 5 μm.	95% for 30 days
MiStent	Micell	PLGA	2–3 months	Evenly distributed around the circumference, the presence of crystalline sirolimus.	DESSOLVE-I (*n* = 30)
Ultimaster	Terumo	Poly (D, L-lactide) and poly(ε-caprolactone)	3–4 months	Positioned on the outer side, extending to a distance of 15 μm.	90% for 90 days

**Table 4:** Characteristics of biodegradable coronary stents.

Type	Producer	Material	Biodegradation Time	Coating Material	Drug Used	Drug Release Period
BVS V.1.0 and BVS V.1.1	Abbott	Poly-L-lactic acid	2 years	PDLLA	Everolimus	Over a 30-day period, there was an 80% occurrence rate
DESolve	Elixir Medical	Poly-L-lactic acid	1–2 years	PLLA	Myolimus	Over a 30-day period, there was an 80% occurrence rate
ReZolve and REVA Gen	REVA Medical	PC	2 years	-	Sirolimus and paclitaxel	-
IDEAL	Xenogenics	Poly-anhydride ester	200	9–12 months	Salicylate	Sirolimus
ART 18Z	Arterial Remodeling Technology	Poly-D,L-lactic acid	18 months	-	-	-
Xinsorb BRS	Huaan Biotechnology	PLLA, PCL, PLGA	-	-	Sirolimus	-
Amaranth BRS	Amaranth Medical	PLLA	1 year	-	-	-
AMS-1 and AMS-2	Biotronik	Mg alloy	<4 months and >4 months resp.	-	-	-
DREAMS-1 and DREAMS-2	Biotronik	Mg alloy	9 months	PLGA and poly-lactic acid	Paclitaxel and sirolimus	-

## Data Availability

No new data were created or analyzed in this study. Data sharing is not applicable to this article.
